# Novel *GANAB* variants associated with polycystic liver disease

**DOI:** 10.1186/s13023-020-01585-4

**Published:** 2020-10-23

**Authors:** Liyanne F. M. van de Laarschot, René H. M. te Morsche, Alexander Hoischen, Hanka Venselaar, Hennie M. Roelofs, Wybrich R. Cnossen, Jesus M. Banales, Ronald Roepman, Joost P. H. Drenth

**Affiliations:** 1 Department of Gastroenterology and Hepatology, Institute for Molecular Life Sciences, Radboud University Medical Center, P.O. Box 9101, 6500 HB Nijmegen, The Netherlands; 2 Department of Human Genetics, Institute for Molecular Life Sciences, Radboud University Medical Center, Nijmegen, The Netherlands; 3 Centre for Molecular and Biomolecular Informatics, Institute for Molecular Life Sciences, Radboud University Medical Center, Nijmegen, The Netherlands; 4 Department of Liver and Gastrointestinal Diseases, Biodonostia Research Institute – Donostia University Hospital, University of the Basque Country (UPV/EHU), San Sebastián, Spain; 5 IKERBASQUE, San Sebastián, Spain; 6 CIBERehd, San Sebastián, Spain

**Keywords:** Polycystic liver disease, Glucosidase II, Molecular inversion probe analysis, Liver cysts, Cholangiocytes

## Abstract

**Background:**

Polycystic liver disease (PLD) is an inherited disorder characterized by numerous cysts in the liver. Autosomal dominant polycystic kidney and liver disease (ADPKD and ADPLD, respectively) have been linked to pathogenic *GANAB* variants. *GANAB* encodes the α-subunit of glucosidase II (GIIα). Here, we report the identification of novel *GANAB* variants in an international cohort of patients with the primary phenotype of PLD using molecular inversion probe analysis.

**Results:**

Five novel *GANAB* variants were identified in a cohort of 625 patients with ADPKD or ADPLD. In silico analysis revealed that these variants are likely to affect functionally important domains of glucosidase II α-subunit. Missense variant c.1835G>C p.(Arg612Pro) was predicted to disrupt the structure of the active site of the protein, likely reducing its activity. Frameshift variant c.687delT p.(Asp229Glufs*60) introduces a premature termination codon predicted to have no activity. Two nonsense variants (c.2509C>T; p.(Arg837*), and c.2656C>T; p.(Arg886*)) and splice variant c.2002+1G>C, which causes aberrant pre-mRNA splicing and affecting RNA processing, result in truncated proteins and are predicted to cause abnormal binding of α- and β-subunits of glucosidase II, thus affecting its enzymatic activity. Analysis of glucosidase II subunits in cell lines shows expression of a truncated GIIα protein in cells with c.687delT, c.2509C>T, c.2656C>T, and c.2002+1G>C variants. Incomplete colocalization of the subunits was present in cells with c.687delT or c.2002+1G>C variants. Other variants showed normal distribution of GIIα protein.

**Conclusions:**

We identified five novel *GANAB* variants associated with PLD in both ADPKD and ADPLD patients supporting a common pathway in cystogenesis. These variants may lead to decreased or complete loss of enzymatic activity of glucosidase II which makes *GANAB* a candidate gene to be screened in patients with an unknown genetic background.

## Background

Development of numerous liver cysts is the primary phenotype of autosomal dominant polycystic liver disease (ADPLD). ADPLD patients may present with few kidney cysts, but renal function remains preserved [1, 2]. On the contrary, kidney cysts are the dominant feature of patients with autosomal dominant polycystic kidney disease (ADPKD), and the majority of these patients develop concurrent liver cysts [3]. Almost all ADPKD patients harbour gene mutations in polycystic kidney disease 1 (*PKD1*) or polycystic kidney disease 2 (*PKD2*) [4]. The genetic landscape in ADPLD is more diverse as, to date, mutations in at least seven genes explain collectively only 25–30% of the genetic spectrum of ADPLD [2, 5]. Although ADPLD and ADPKD are two distinct genetic disorders, they share PLD as a major phenotypic feature. Recent evidence suggests that the involved proteins interact, with decreased levels of functional polycystin-1 (PC1), encoded by *PKD1*, as the central element for cyst development [6, 7].

With the exception of LRP5 (a transmembrane protein part of the LRP5/LRP6/Frizzled co-receptor complex [8, 9]) all genes involved in ADPLD encode proteins involved in endoplasmic reticulum trafficking and quality control of glycoproteins. These genes include *PRKCSH*, *SEC63*, *GANAB*, *ALG8*, *SEC61B*, *DNAJB11*, and *ALG9* [10]. Central to the pathomechanism of ADPLD is glucosidase II, an endoplasmic reticulum (ER) resident N-linked glycan-processing enzyme. Glucosidase II (GII) is a complex of catalytic α-subunit (GIIα), encoded by *GANAB*, and a regulatory β-subunit (GIIβ), encoded by *PRKCSH*. Collectively this contributes to the localization and enzymatic activity of GIIα [11]. Glucosidase II catalyzes the two-step hydrolysis at α1,3-linked glucose–glucose and glucose–mannose residues of high-mannose-type glycans to generate a quality control protein tag on glycoproteins that is recognized by ER chaperones [12]. As such, it acts as a major partner in glycoprotein processing and quality control in the ER [13].

The concept that genetic defects disrupting different subunits of a protein complex result in a very similar phenotype (“guilt by association”) is well-accepted [14]. While mutations in GIIβ have been associated with ADPLD, the phenotypic picture for GIIα is less clear. Most studies have linked *GANAB* to ADPKD and renal cysts were present in most patients [15]. We set out to explore the incidence of *GANAB* mutations in a large, independent cohort of patients with PLD as dominant phenotype, and to evaluate the effect of *GANAB* mutations on protein structure and glucosidase II subunit binding.

## Results

### Patient characteristics

For our molecular inversion probe (MIP) analysis we included a total of 625 patients with ADPKD or ADPLD. Patients were referred to our center for genetic analysis or treatment of PLD by hospitals from the Netherlands, Belgium, Spain, and Denmark. All patients had the primary phenotype of polycystic liver disease (PLD). Patients were diagnosed with ADPKD or ADPLD based on clinical presentation. 90 patients were diagnosed with ADPKD, and 535 patients with ADPLD. Of the 625 analysed patients, 17 families were included that consisted of 62 family members. Patients had not been genetically screened previously.

Of the eight patients with likely pathogenic heterozygous *GANAB* variants, seven patients presented with ADPLD and three patients had 1–3 kidney cysts, while in the remaining patients no kidney cysts could be detected. One patient was diagnosed with ADPKD. The hepatic phenotype did not differ between patients affected by ADPLD or ADPKD. Six out of eight patients were female and the mean age was 56 (range 31–79) years. Clinical presentation of patients with *GANAB* variants can be found in Table 1.

**Table 1:** Clinical presentation of kidney and liver disease in 8 affected individuals with *GANAB* variants

	Family 11-0741		Unrelated individuals					
	8951	9087	8380	11726	11475	11716	8700	12241
Gender	Female	Female	Male	Female	Male	Female	Female	Female
Diagnosis	ADPLD	ADPLD	ADPLD	ADPLD	ADPLD	ADPKD	ADPLD	ADPLD
*Mutation* (*RefSeq NM_198335*)								
Chromosome position (Hg19)	g.62400926TG>G	g.62400926TG>G	g.62393841C>T	g.62394111C>T	g.62396665G>C	g.62414056delTAGCGG	g.62397125G>C	g.62397125G>C
cDNA change	c.687delT	c.687delT	c.2656C>T	c.2509C>T	c.2002+1G>C	c.11_16delTAGCGG	c.1835G>C	c.1835G>C
Amino acid change	p.Asp229Glufs*60	p.Asp229Glufs*60	p.Arg886*	p.Arg837*	Splicing variant	p.Val4_Ala5del	p.Arg612Pro	p.Arg612Pro
*Radiological presentation*								
Type	CT	Ultrasound	CT	CT	n/a	MRI	CT	CT
Liver cysts	> 20 cysts	multiple cysts	15 cysts	> 20 cysts	n/a	> 20 cysts	> 10 cysts	> 30 cysts
kidney cysts	No	No	2 bilateral cysts	No	n/a	> 20 bilateral cysts	3 cysts in left kidney	1 cyst in left kidney

*ADPKD* autosomal dominant polycystic kidney disease, *ADPLD* autosomal dominant polycystic liver disease, *CT* computed tomography, *MRI* magnetic resonance imaging, *n/a* not available for review

### Identification of GANAB variants in PLD-affected individuals and families

**Fig. 1:**
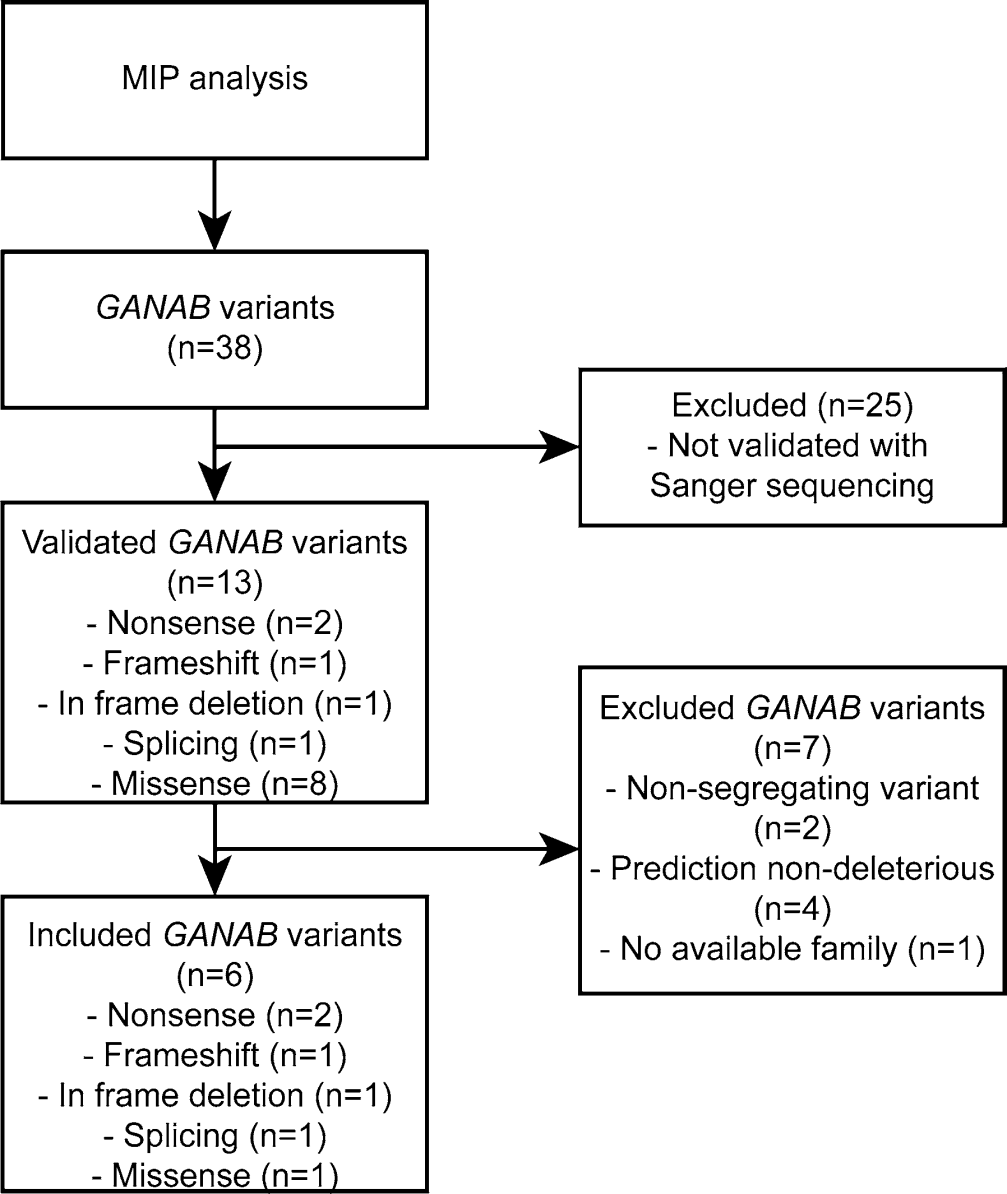
Flow chart of the MIP analysis. Of the 625 polycystic liver patients included, we identified 32 patients with 38 *GANAB* variants. 13 of these variants were validated using Sanger sequencing. The non-validated variants were excluded from further analysis. Of the validated variants, 7 were excluded because these were non-segregating, not predicted to be deleterious by in silico prediction tools, or no family members were available for further co-segregation analysis. 6 variants were included as definitive pathogenic *GANAB* variants

MIP analysis identified 38 variants in 32 patients, of which 13 heterozygous *GANAB* variants are possibly pathogenic. We identified one frameshift, one in-frame deletion, one splice site, two nonsense, and eight missense variants that were all validated by Sanger sequencing (Fig. 1, Additional File 1). Notably, none of these variants have been reported before to cause ADPLD or ADPKD [15]. Moreover, registry data show that in-frame deletion c.11_16delTAGCGG (rs750723025) has a global frequency of 9/249,084 exomes (MAF = 0.000036; GnomAD) and is seen in 1/133,716 European exomes (MAF = 0.000007). The c.2656C>T variant (rs1210158408) is detected in 1/251,486 exomes (MAF = 0.000003976; GnomAD). The c.323C>T variant (rs200232092) is detected in 7/2,541,458 exomes (MAF = 0.000028; GnomAD). The other variants are previously unknown and no allele frequency is available.

### Segregation analysis of GANAB variants

To bolster the causal relationship between *GANAB* variants identified by MIP analysis and clinical presentation of PLD, we tested DNA of family members for *GANAB* variants. We confirmed that frameshift variant c.687delT co-segregated with the disease in family 11-0741. This ADPLD family presented with multiple liver cysts, without renal cysts which suggests that the variant is likely pathogenic.

For the patients with splice variant (c.2002+1G>C), and nonsense variants (c.2509C>T and c.2656C>T) no DNA of family members was available for segregation analysis. Due to the predicted severe nature of these variants we included them in our final group of likely pathogenic variants.

Of the 8 missense variants, four variants (c.1852C>T, c.2006A>G, c.38G>A, c.1835G>C) were predicted as deleterious by four distinct in silico predictors.

We analyzed two missense variants (c.1852C>T and c.2006A>G) predicted as deleterious, for segregation in affected family members. Missense variant c.1852C>T was present in the affected individual and her unaffected daughter. We did not identify a pathogenic *GANAB* variant in DNA derived from the affected brother of affected individual nor were mutations in other known genes for ADPLD present. Similarly, for the individual having the c.2006A>G mutation both affected and unaffected family members possessed the *GANAB* variant. This suggests that neither variant c.1852C>T nor c.2006A>G are causative for PLD in these families.

For the remaining two missense variants (c.38G>A, c.1835G>C) predicted as deleterious no family members were available for further analysis. However variant c.1835G>C p.(Arg612Pro) was reported in two unrelated individuals and is therefore included in the group of likely pathogenic variants.

Three missense variants (c.323C>T, c.1607A>C, c.1883C>G) are predicted to be probably deleterious, the other variant (c.2702C>T) is predicted to be not deleterious by in silico prediction tools. These four variants were discarded as bona fide mutations due to the lack of available family members and the results of the prediction tools.

### MIP analysis of PRKCSH, SEC63 and PKD2

We analyzed all patients with a validated likely pathogenic *GANAB* variant for a pathogenic sequence variation in *PRKCSH*, *SEC63* and *PKD2*. None of the individuals who carried a bona fide pathogenic *GANAB* variant was trans heterozygous for any of the other three PLD associated genes. In the family with non-segregating *GANAB* variant c.2006A>G, we identified the *PRKCSH* variant c.841C>T p.(Arg281Trp), which has previously been described to cause APDLD [16].

### Pathogenic GANAB variants influence glucosidase II structure in silico

**Fig. 2:**
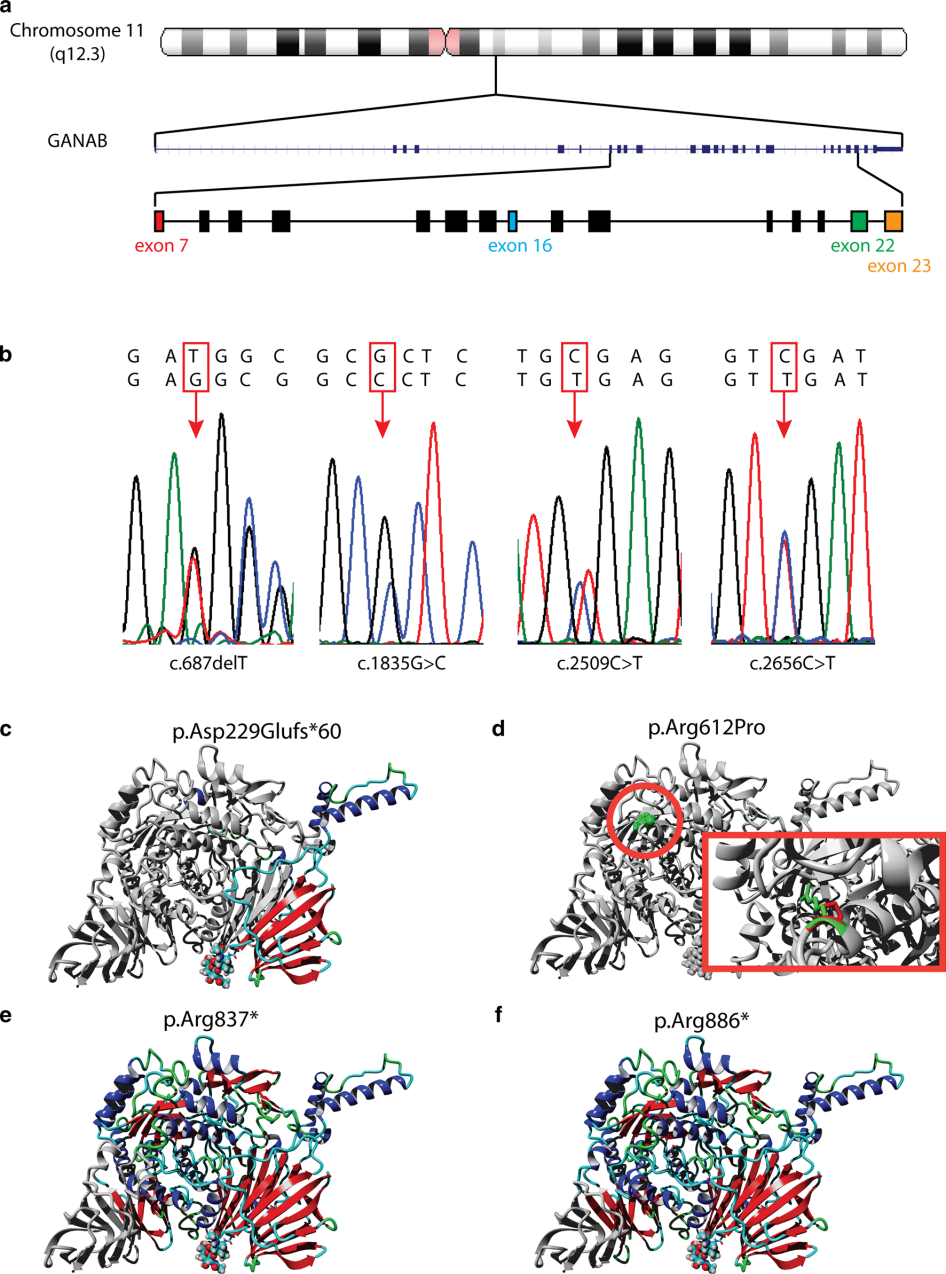
Identification of *GANAB* variants and in silico prediction of effect of variants on GIIα structure. **a** *GANAB* is located on chromosome 13q12.3 and consists of 25 exons. The identified variants are located on exons 7, 16, 22, and 23. **b** Sequence electropherograms show heterozygous germline mutations **c** Predicted structure with p.Asp229Glufs*60 (c.687delT) variant resulting in early termination of the protein by introducing a premature stop codon. **d** p.Arg612Pro (c.1835G>C) variant results in amino acid change close to the active site of the protein. Affected amino acid is depicted in color. The inlay shows a magnification of the structural change of the amino acid. **e** Predicted structure with c.2509C>T (p.Arg837*) variant results in untranscribed distal C-terminal domain **f** Predicted structure with c.2656C>T (p.Arg886*) variant results in untranscribed distal C-terminal domain. **c**, **e**, **f** Preserved protein is depicted in color, loss of protein structure in grey

In order to predict the functional consequences of the identified *GANAB* variants, we evaluated their effects on the glucosidase II protein complex structure in silico. The relationship between the *GANAB* variants and the GII function and interaction of GII subunits was studied using 3D homology modelling of the GII protein complex. Structural changes caused by the *GANAB* variants were predicted and visualized. The complete protein structure and interaction sites of the two subunits of human GII have only recently been unraveled [12, 17]. The GII protein complex consists of the 110 kDA catalytic α-subunit and the 60 kDA regulatory β-subunit. GIIα is composed of four major domains and three subdomains. The active site of GIIα can be found in the β_8_α_8_ barrel domain [12]. The distal C-terminal domain of GIIα is primarily involved in binding of GIIβ through its N-terminal GIIα-binding domain [17]. The frameshift variant (c.687delT) is located at the N-terminal domain. Modelling predicts that this variant introduces a premature termination codon (PTC) 60 amino acids upstream (p.Asp229Glufs*60) leading to a truncated protein and thus limiting functional protein expression. In frame deletion p.Val4_Ala5del (c.11_16delTAGCGG) located to the signalling sequence, is predicted to result in degradation of the protein as the protein would be unable to be transported and mature. The amino acid substitution p.Arg612Pro in the β_8_α_8_ barrel domain is the consequence of missense variant c.1835G>C. This affects the structure of the domain carrying the active site of GIIα and thus is predicted to reduce its activity. The two nonsense variants p.Arg837* (c.2509C>T) and p.Arg886* (c.2656C>T) result in a truncation of the distal C-terminal domain (Fig. 2). The last variant is located in a splice site region at the distal part of the gene, which is predicted to result in inaccurate pre-mRNA splicing. Most likely this leads to a PTC resulting in a truncated protein.

### Glucosidase II α-subunit expression and localization in HEK293 and HeLa cells

**Fig. 3:**
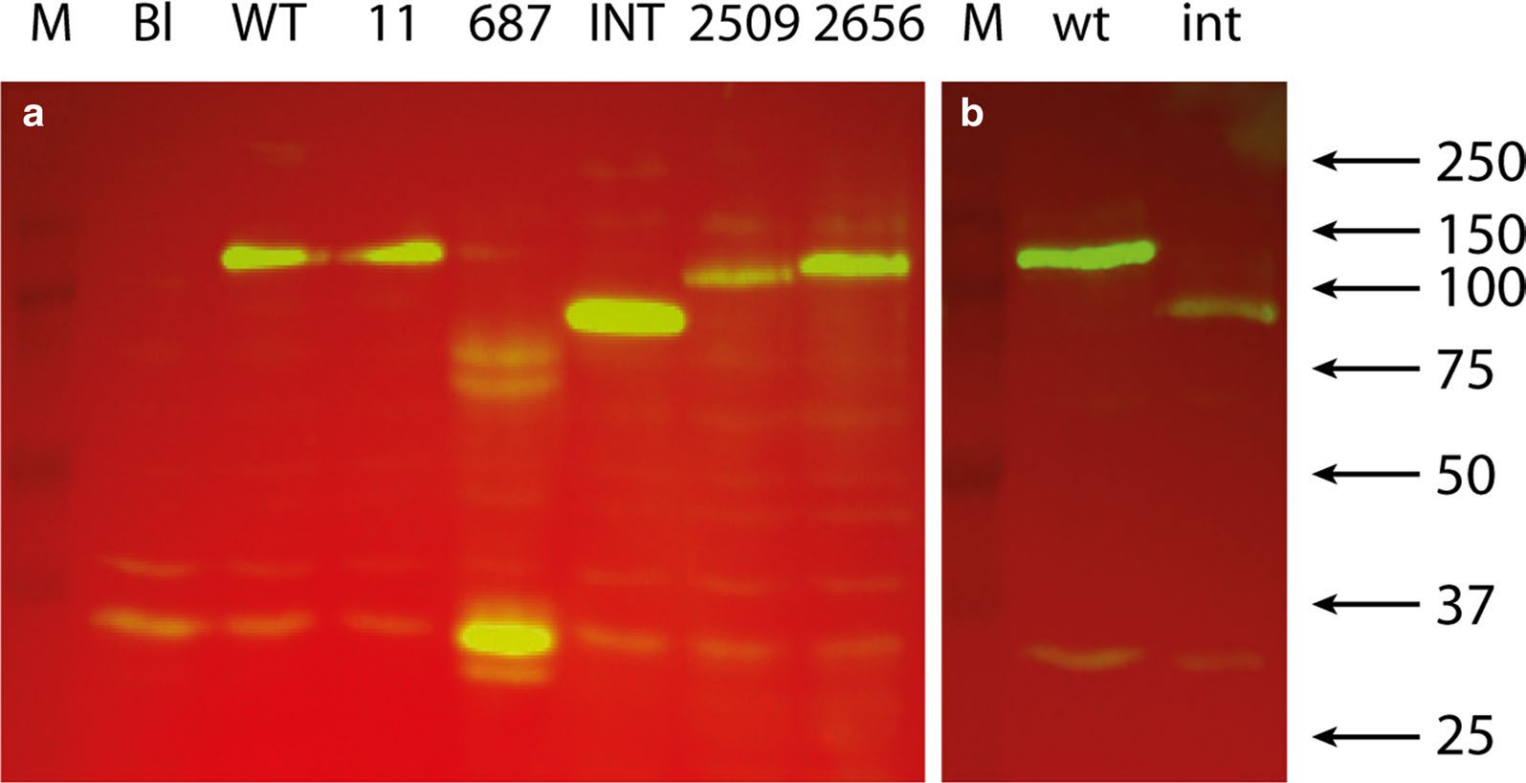
Protein analysis of wild type and mutant GIIα in HEK293 cells. Western blot detection using anti-Flag antibody of cell lysate from transiently transfected HEK293 cells using N-terminal Flag tagged constructs with: **a** *GANAB* Wild Type (WT) and variants c.11_16delTAGCGG (11), c.687delT (687) and c.2002+1G>C (INT) at te left of the marker (M). The constructs showed similar amounts of expressed GIIα with predicted molecular weights; **b** stable transfected HEK293 cells resulted in reduced level of truncated GIIα (INT) compared to the normal GIIα (WT) using the intron construct

We transiently transfected the *GANAB* Wild Type (*GANAB-WT*) and the suspected pathogenic variants c.11_16delTAGCGG (*GANAB-11*), c.687delT (*GANAB-687*), c.2002+1G>C (*GANAB-INT*), c.2509C>T (*GANAB-2509*) and c.2656C>T (*GANAB-2656*) constructs in HEK293 and HeLa cells. Western blots using equal amounts of total protein from transiently transfected cells revealed expression of GIIα, truncated and non-truncated in HEK293 cells (Fig. 3a). *GANAB-11* generated GIIα with a normal appearing length. *GANAB-687* resulted in truncated GIIα of about 32 kDa which is in line with the prediction of a 287 amino acid long truncated protein. *GANAB-INT* produced a truncated protein of about 80 kDa suggesting the use of a stop codon starting 51 bp upstream of exon 17. There were no quantitative expression differences which suggests there may be no nonsense-mediated RNA decay provoked by the presence of a PTC in variants *GANAB-687* and *GANAB-INT. GANAB-2509* resulted in truncated GIIα of about 92 kDa and *GANAB-2656* in a a truncated protein of about 97 kDa. This is in line with the size of the predicted truncated protein. Experiments using HeLa cells produced similar results (Additional File 2). On the other hand, protein analysis of cells stably expressing the wild type and mutated intronic variant showed a 50% reduction of the truncated protein in cells (Fig. 3b) with an equal copy number of the incorporated gene (Additional File 3).

**Fig. 4:**
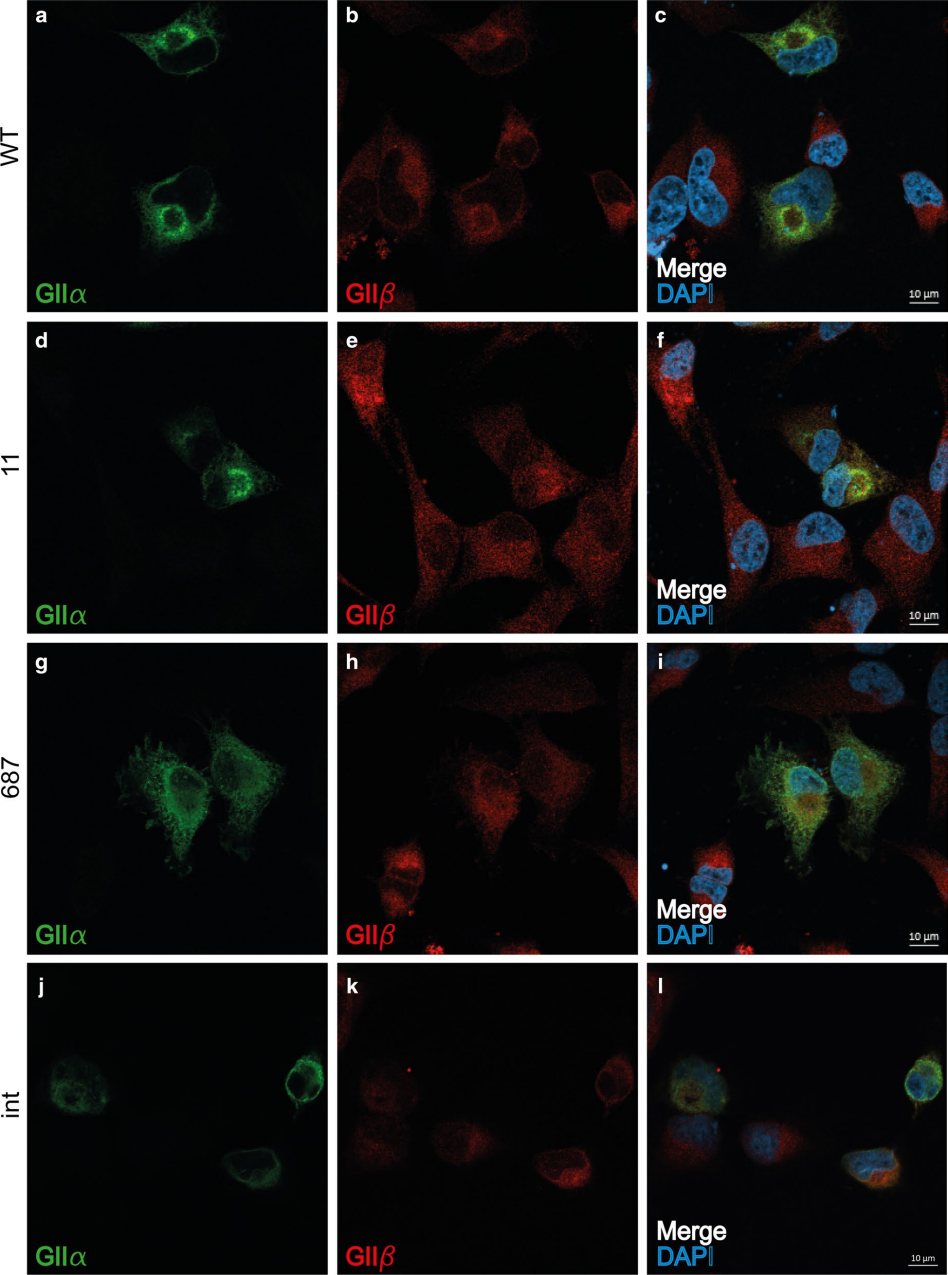
Localization of wild-type and mutant GIIα in HeLa cells. Immunofluorescence staining of HeLa cells transfected with *GANAB* Wild Type (WT; **a**–**c**) and variants c.11_16delTAGCGG (11; **d**–**f**), c.687delT (687; **g**–**i**) and c.2002+1G>C (int; **j**–**l**). GIIα (green) staining shows co-localization to GIIβ (red) with WT and variant 11 where variant 687 shows localization throughout the cell and splice variant int localizes to the nucleus (blue)

Immunofluorescent images of HeLa cells transfected with *GANAB-WT* showed normal distribution of GIIα and co-localization with GIIβ (Fig. 4a–c). Also, cells transfected with *GANAB-11* showed a normal distribution of GIIα and co-localization with GIIβ which was in contrast to the predicted in silico analysis (Fig. 4d–f). Cells transfected with *GANAB-687* (Fig. 4g–i) and *GANAB-INT* (Fig. 4j–l) demonstrated abberant GIIα localization distributed throughout the cell or confined to the nucleus respectively. Co-localization with GIIβ was hardly present probably due to absence of the C-terminal part of GIIα. Cells transfected with *GANAB-2509* or *GANAB-2656* showed normal distribution of GIIα despite the predicted partial absence of the C-terminal part of GIIα (Additional File 4).

## Discussion

Here we describe 5 novel *GANAB* variants that can be linked to the presence of PLD [15]. All patients presented severe PLD. Three ADPLD patients possessed 1–3 non-pathogenic kidney cysts, which is in concordance with the prevalence of kidney cysts in the general population [18]. These variants can cause PLD in patients diagnosed with ADPLD or ADPKD. Seven out of eight patients with a confirmed pathogenic *GANAB* variation in our cohort were diagnosed with ADPLD, the last patient had ADPKD.

Our in silico analysis suggests that the amino acid substitution in the β_8_α_8_ barrel domain p.(Arg612Pro) probably results in reduced GIIα enzymatic activity because the active site domain of the subunit is predicted to be disrupted. The variants c.2656C>T p.(Arg886*) and c.2509C>T p.(Arg837*) introduce a PTC that would lead to truncation of the C-terminal domain of GIIα. This could impair interaction with GIIβ and consequently decrease enzymatic activity of GIIα [17, 19]. This is in line with an interaction study showing that the Arg834Ala/Arg835Ala mutation impairs interaction between GIIα and GIIβ subunits [17]. Further, from genetic studies the missense mutation in the distal C-terminal p.Arg839trp was identified to cause ADPKD in an ADPKD family [15]. ER-localization of GIIα is mediated by GIIβ [20]. Our immunofluorescence assay did not show subcellular localization of GIIα. This would imply that variants c.2656C>T and c.2509C>T do not impair GIIα and GIIβ co-localization but may still affect enzymatic activity of GIIα.

The frameshift variant c.687delT p.(Asp229Glufs*60) and the splice variant c.2002+1G>C are likely to result in truncated proteins lacking the C-terminal domain of GIIα and can result in nonsense-mediated mRNA decay that reduces the level of translated protein expressed in the ER. However, no reduced levels of truncated proteins were seen in the transiently transfected cells compared to the wild type suggesting no influence of the PTCs on nonsense-mediated mRNA decay. Because transient transfection of cells can sometimes influence splicing and normal cellular processes due to overexpression of proteins we also performed stable transfection of the variants in which a reduced level of truncated protein of *GANAB-INT* was observed and therefore possibly indicates some, but not complete nonsense-mediated mRNA decay. Immunofluorescence assay shows no co-localization of both variants with GIIβ and thus incomplete interaction which leads to reduced enzymatic of the GII complex.

Our study knows some limitations. Our study yielded a relative high number of non-validated *GANAB* variants. This was because all variants with a quality of depth of 500 or higher were included. This lower quality setting was chosen in order to not oversee any bona fide pathogenic variants in *GANAB* or the other three PLD genes. All cell studies were performed in HEK293 and HeLa cell lines. These cell lines are not specifically used as PLD models. Since the aim of our study was not to model PLD, but to study protein expression and localization of GII protein complex, these cell lines were chosen for their expression of both *PRKCSH* and *GANAB.* Cholangiocyte cell lines such as H69 or SkCHA-1 may be closer related to affected cells in PLD patients, but have low transfection rate and were thus deemed less suitable for our studies.

Overall, most identified variants will likely lead to decreased or complete loss of functional glucosidase II. Trimming of N-glycan glucose residues by glucosidase II is necessary for recognition by ER-resident chaperone proteins calnexin and calreticulin, and subsequent protein folding and secretion [21]. Therefore, functionality of glucosidase II is essential for correct protein maturation. We can hypothesize that defective glucosidase II directly affects polycystin-1 folding and maturation, and in this way causes cyst formation in the liver and/or kidney. However, as glucosidase II-driven protein maturation is not limited to polycystin-1, the absence of disease symptoms due to other disrupted cellular functions or organs remains unexplained. Also, it remains unclear what factors contribute to the differential expression of cysts in the liver and/or kidney. This should be investigated in future studies.

## Conclusions

Our findings have two important implications. First, *GANAB* is the only gene that has been shown to cause polycystic liver disease in patients with ADPLD or ADPKD. This finding supports the standing hypothesis of a common pathway of hepatic cystogenesis in patients with ADPLD and ADPKD [22]. In vitro studies showed that mutations in genes associated with ADPLD result in defective maturation and trafficking of polycystin-1 [7, 15]. The level of functional polycystin-1 at the cilium is postulated to be central in the development of both hepatic and kidney cysts [10]. If the level of functional polycystin-1 drops below a critical threshold, cyst development initiates. Furthermore, a more severe reduction of polycystin-1 function causes more severe cystic disease [6, 23]. Maturation of polycystin-1 is dependent on polycystin-2 chaperone function [24]. Moreover, the abundance of polycystin-2 is determined by N-glycosylation mediated by glucosidase II [25].

Second, our findings have some implications for clinical practice. Our cohort contains a large number of unrelated patients from different countries in Europe, which indicates that our results are a good reflection of the prevalence of *GANAB* variants in the European PLD patient population. In our study population the detection rate of bona fide pathogenic *GANAB* variants is ~ 1%. The detection rate in our total group of PLD-affected patients is low, nonetheless this number is similar to the detection rate of ADPLD associated gene *LRP5* [9]. We suggest that genetic screening should include a screen for *GANAB* variants in ADPLD and ADPKD patients with unknown genetic background.

To conclude, we identified five novel *GANAB* variants associated with PLD in ADPKD and ADPLD patients. These variants may affect functionally important domains of GIIα and lead to decreased or complete loss of enzymatic activity of glucosidase II.

## Methods

### Sample and data collection

We collected clinical data and biomaterials from PLD patients in our PLD registry [26]. All participants of the PLD cohort provided informed consent for DNA analysis related to PLD studies. Blood samples were collected from patients and all available family members. Genomic DNA was isolated from the blood samples by standard methods with High Pure PCR Template Kit (Roche, Basel, Switzerland). Clinical and imaging data were obtained through review of clinical records.

### Editorial policies and ethical considerations

This study was performed in line with the principles of the Declaration of Helsinki. Approval was granted by the Ethics Committee of the Radboud University Medical Center (CMO regio Arnhem-Nijmegen; 2001–218) and all participants gave informed consent.

### Molecular inversion probe analysis

We used molecular inversion probe (MIP) analysis for DNA analysis of *GANAB* (RefSeq NM_198335), *PRKCSH* (RefSeq NM_001289104), *SEC63* (RefSeq NM_007214), and *PKD2* (RefSeq NM_000297)*. PRKCSH, SEC63,* and *PKD2* were included in this analysis to exclude that PLD was caused by any of the known genes in these individuals. Due to technical difficulties caused by genomic duplicates in the first 34 exons, *PKD1* was not included in the final MIP library. The final pooled MIP library comprised 174 probes (*GANAB* n = 54, *PRKCSH* n = 33, *SEC63* n = 45, *PKD2* n = 42). The average coverage per probe was *GANAB* 1277, *PRKCSH* 263, *SEC63* 1369, *PKD2* 759. Capture regions were sequenced in two runs using 112-bp reads on a NextSeq500 sequencer (Illumina). MIP data variants were identified as described before [27]. Briefly, reads were aligned by Burrows-Wheeler Aligner to the reference genome. After MIP extension and ligation arms were removed from all the alignment files, reads were trimmed to remove overlap between the paired-ends. The identified variants were filtered on gene component (exon, acceptor site canonical, splice donor site canonical), synonymous (false), and zygosity (heterozygote) to exclude all false positive and non-changing variants. Variants that passed all filtering steps were validated using Sanger sequencing.

### Sanger sequencing

Primers (Sigma-Aldrich, St Louis, MO) to amplify *GANAB* (25 exons), *PRKCSH* (18 exons), *SEC63* (21 exons) and *PKD2* (15 exons) were designed using online software Primer3 (https://primer3.ut.ee/), and were used for PCR amplification (TL100 Biorad, Hercules, CA) of DNA samples. DNA bands were cut out of 1.5% agarose gel and purified using the QIAEXII kit (Qiagen, Hilden, Germany). Next, 10 ng DNA per sample was used for Sanger sequencing. Sanger sequencing was performed at the Radboudumc Sequence Facility according to standard Sanger methods (3730 DNA Analyzer (Applied Biosystems, Foster City, CA)) with support of Big Dye Terminator ( v1.1 Thermofisher Scientific, Waltham, MA)).

Variants found with MIP analysis were compared to the obtained Sanger sequences with Alamut Visual (v2.7). The functional significance of novel mutations was assessed with bioinformatics prediction tools (PolyPhen-2, Align GVGD, SIFT, MutationTaster). Sanger sequencing was performed in all available family members for those patients with validated mutations. Validated variants were submitted to ClinVar database.

### In Silico analysis and homology modeling

Homology models of the human GIIα were created using the experimentally solved 3D-structure of the murine GIIα as a template (Protein Data Bank file 5F0E) [28]. The human and mouse sequences show 92% sequence identity over 851 amino acids. The models were created using the automatic modelling script with standard parameters of the YASARA & WHAT IF Twinset [29, 30]. The resulting model is complete, except for the N-terminal signal peptide.

### GANAB constructs

*GANAB* expression clones were generated using the Gateway Cloning system (Thermo Scientific, Carlsbad, CA). Entry clone encoding full-length human *GANAB* (2901 bp) was generated from cDNA of liver tissue. Total RNA was isolated from liver tissue using TRIzol Reagent (Thermo Scientific), and cDNA was generated by RT Transcriptor First Strand cDNA synthesis kit (Roche Applied Sciences, Mannheim, Germany) using a *GANAB* specific reverse primer. Full-length *GANAB-WT* fragments were obtained using Platinum™ SuperFi™ DNA Polymerase (Thermo Scientific) using forward and reverse primers including the attB1 and attB2 sequence. The *GANAB-WT* fragment was then cloned into the Gateway entry vector pDONR201. The entry clones of *GANAB* including wild type and mutant intron 17 were generated by replacing a BglI/KpnI fragment of pDONR201*GANAB-WT* by a fragment produced by PCR on patient genomic DNA having a heterozygous intronic mutation using an exon 14 forward and exon 18 reverse primer followed by digestion with BglI and KpnI. All *GANAB* entry clones were subsequently cloned into the GW331 N-terminal FLAG expression vector. *GANAB* constructs harboring exonic variants were generated using the Quick Change-II-XL Site-Directed Mutagenesis Kit (Agilent Technologies, Santa Clara, USA) and the GW331*GANAB-WT* as a template. Sequences of all constructs were confirmed by Sanger sequencing.

### Transfection of cells

HEK293 and HeLa cells were cultured in DMEM supplemented with 10% FCS, 1% NEAA, 50 µg/mL Gentamicin, and 10 mM HEPES (all Thermo Scientific) at 37 °C and 5% CO_2_ in a humidified incubator. For DNA transfections, HEK293 and HeLa cells were seeded in a 6-wells plate (500,000 cells per well) for Western blotting and HeLa cells also in a 24-wells plate containing poly-l-lysine coated glasses (100,000 cells per well) for immunofluorescent staining. The following day cells were transfected with GW331*GANAB* constructs using FuGene HD (Promega, Madison, WI). For stable expression of GIIα, normal medium of cells was replaced by medium containing 500 µg/ mL G418 (Thermo Scientific) after 2 days. After 2 weeks resistant clones were transferred to a new well and tested for stable expression of GIIα by Western blotting.

### Immunofluorescent staining

Human cholangiocytes or HeLa cells were fixed in 2% paraformaldehyde and incubated in phosphate buffered saline/1% Bovine serum albumin/1% Normal swine serum/0.1% Cold water fish gelatin containing primary antibodies against PRKCSH (1:100, mouse polyclonal, Santa Cruz Biotechnology, Dallas, TX), GANAB (1:200, rabbit polyclonal, Thermofisher Scientific), FLAG (1:100, rabbit polyclonal, Sigma) or protein disulfide isomerase (PDI, 1:100 mouse monoclonal, Stressgen Biotechnologies, San Diego, CA). Then glasses were incubated with the corresponding secondary fluorescent antibodies Texas Red (1:100, mouse, Jackson Immunoresearch, West Grove, PA), FITC (1:100, rabbit, Jackson Immunoresearch) and DAPI (1:1000, Roche) for nuclear staining. Images were taken using FV1000 Confocal Laser Scanning Microscope (Olympus, Tokio, Japan).

### Western blot analysis

Cells were lysed using lysis buffer (50 mm Tris–HCl (pH7.5), 150 mm NaCl, 1% NP-40, protease inhibitor tablet (Roche)) on ice 72 h after transfection. After adding sample buffer to the lysed cells, samples were heated for 5 min at 95 °C. Samples were run on a 10% SDS-PAGE gel and transferred semi dry onto a nitrocellulose membrane. Immunostaining was perfomed with anti-Flag as primary antibody and swine anti-rabbit HRP as a secondary antibody (DAKO, Glostrup, Denmark). Proteins were visualized using Clarity Western ECL Substrate (Biorad) and the Proxima C18 imaging system (Isogen, de Maarn, Netherlands). Western Blots were quantified using Totallab quant (Totallab, Newcastle-Upon-Tyne, United Kingdom).

### RNA isolation and RT-qPCR

Total RNA was isolated using TRIzol according to manufacturer’s protocol. We reverse transcribed RNA into complementary DNA (cDNA) using iScript cDNA synthesis kit according to the protocol (Biorad). 1 mL of resulting cDNA was used for RT-qPCR. Briefly, the RT-qPCR was carried out on an thermal cycler (CFX96, Biorad), using the 2_DDCt SYBR green protocol. We amplified GANAB and beta actin (reference) in 40 cycles. RT-qPCR data were analyzed using the CFX-Manager software, which validates primer quality by analyzing melting curves. All the RT-qPCRs were performed with triplicates for each sample.

## Supplementary information

**Additional file 1**. Overview of coverage and Sanger sequencing results of 38 GANAB variants identified by molecular inversion probe analysis

**Additional file 2**. Protein analysis of wild type and mutant GIIα in HeLa cells. Western blot detection using anti-Flag antibody of cell lysate from transiently transfected HeLa cells using N-terminal Flag tagged construct with GANAB Wild Type (WT) and variants c.11_16delTAGCGG (11), c.687delT (687), c.2002+1G>C (INT), c.2509C>T (2509) and c.2656C>T (2656) at the left of the marker (M). The constructs showed similar amounts of expressed GIIα with predicted molecular weights

**Additional file 3**. DNA and protein expression analysis of GIIα. DNA expression of GIIα Wild Type intron (WT) and mutant intron (MT intron) was analysed using qPCR with B-actin (ACTB) as reference. WT expression was 1.46 times higher than MT intron. On Western Blot expression of WT protein was 4 times higher than MT intron protein. In absolute numbers protein expression of WT intron was 2.67 times higher than MT intron protein

**Additional file 4**. Localization of wild-type and mutant GIIα in HeLa cells. Immunofluorescence staining of cells transfected with GANAB Wild Type (WT) and variants c.11_16delTAGCGG (11), c.687delT (687), c.2002+1G>C (int), c.2509C>T (2509), and c.2626C>T (2656). GIIα (green) staining shows normal localization of WT, variant 11, variant 2509, and variant 2656. Variant 687 shows subcellular localization. Variant int localizes to the nucleus (blue), or localizes subcellular

## Data Availability

All new variations found in this study were submitted to the ClinVar database with numbers RCV000758153 to RCV000758162.

## Abbreviations

ADPKD: Autosomal dominant polycystic kidney disease
ADPLD: Autosomal dominant polycystic liver disease
ER: Endoplasmic reticulum
GII: Glucosidase II
GIIα: Glucosidase II α-subunit
GIIβ: Glucosidase II β-subunite
MIP: Molecular inversion probe
PC1: Polycystin-1
PKD1: Polycystic kidney disease 1
PKD2: Polycystic kidney disease 2
PLD: Polycystic liver diseaseα
PTC: Premature termination codon
