# Supplementary material for: Novel GANAB variants associated with polycystic liver disease
Source: Orphanet J Rare Dis. 2020 Oct 23;15:302. doi: 10.1186/s13023-020-01585-4 (PMC7585303; doi:10.1186/s13023-020-01585-4)
Supplement: Supplementary file 1 — Additional file 1. Overview of coverage and Sanger sequencing results of 38 GANAB variants identified by molecular inversion probe analysis [file 13023_2020_1585_MOESM1_ESM.docx]

**Additional File 1** Overview of coverage and Sanger sequencing results of 38 *GANAB* variants identified by molecular inversion probe analysis.

| **Patient ID** | **Coverage (Mutation reads/total reads)** | **Sanger sequencing** | **DNA change** | **Protein change** |
| --- | --- | --- | --- | --- |
| 8951 | 411/917 | Yes | c.687del | p.(Asp229Glufs*60) |
| 8380 | 148/526 | Yes | c.2656C>T | p.(Arg886*) |
| 11726 | 61/172 | Yes | c.2509C>T | p.(Arg837*) |
| 11475 | 358/704 | Yes | c.2002+1G>C | splicing |
| 8700 | 308/517 | Yes | c.1835G>C | p.(Arg612Pro) |
| 8515 | 27/101 | Yes | c.1174C>G | p.(Pro392Ala) |
| 1966 | 10/31 | No | c.2717A>G | p.(Glu906Gly) |
| 8687 | 1251/2632 | Yes | c.2702C>T | p.(Pro901Leu) |
| 9324 | 7/13 | No | c.2582C>T | p.(Thr861Ile) |
| 3805 | 5/20 | No | c.2577+1G>A | splicing |
| 7902 | 15/57 | No | c.2543A>G | p.(Asp848Gly) |
| 8854 | 1/1 | No | c.2543A>G | p.(Asp848Gly) |
| 8531 | 15/73 | No | c.2519G>A | p.(Arg840Gln) |
| 8783 | 19/20 | No | c.2507T>C | p.(Met836Thr) |
| 8043 | 9/38 | No | c.2438T>C | p.(Leu813Pro) |
| 1807 | 11/32 | No | c.2351C>T | p.(Ala784Val) |
| 3848 | 17/82 | No | c.2182T>C | p.(Ser728Pro) |
| 3308 | 83/181 | Yes | c.2006A>G | p.(Asp669Gly) |
| 11433 | 45/176 | No | c.1991C>T | p.(Ser664Phe) |
| 6871 | 75/174 | Yes | c.1883C>G | p.(Ala628Gly) |
| 9167 | 178/443 | Yes | c.1852C>T | p.(Arg618Cys) |
| 8754 | 260/584 | Yes | c.1835G>C | p.(Arg612Pro) |
| 11420 | 10/10 | No | c.1613C>T | p.(Thr538Met) |
| 11206 | 792/1551 | Yes | c.1607A>C | p.(Asn536Thr) |
| 8346 | 5/30 | No | c.1452+2T>C | splicing |
| 7378 | 7/28 | No | c.1447C>T | p.(Arg483Trp |
| 7222 | 6/13 | No | c.1349T>C | p.(Leu450Pro) |
| 6583 | 8/37 | No | c.1340T>C | p.(Val447Ala) |
| 3805 | 6/23 | No | c.1339G>A | p.(Val447Ile) |
| 8515 | 27/101 | No | c.1171_1172insT | p.(Gly391Valfs*6) |
| 8401 | 35/108 | No | c.1120G>A | p.(Asp374Asn) |
| 8043 | 14/68 | No | c.1076A>G | p.(Lys359Arg) |
| 3375 | 2494/4908 | Yes | c.323C>T | p.(Pro108Leu) |
| 10134 | 15/30 | No | c.266T>C | p.(Leu89Pro) |
| 8783 | 7/28 | No | c.191T>C | p.(Leu64Pro) |
| 11402 | 20/46 | Yes | c.38G>A | p.(Arg13Gln) |
| 8783 | 12/26 | No | c.22G>T | p.(Ala8Ser) |
| 11716 | 87/214 | Yes | c.11_16del | p.(Val4_Ala5del) |
