# Supplementary figures and images for: Novel GANAB variants associated with polycystic liver disease
Source: Orphanet J Rare Dis. 2020 Oct 23;15:302. doi: 10.1186/s13023-020-01585-4 (PMC7585303; doi:10.1186/s13023-020-01585-4)

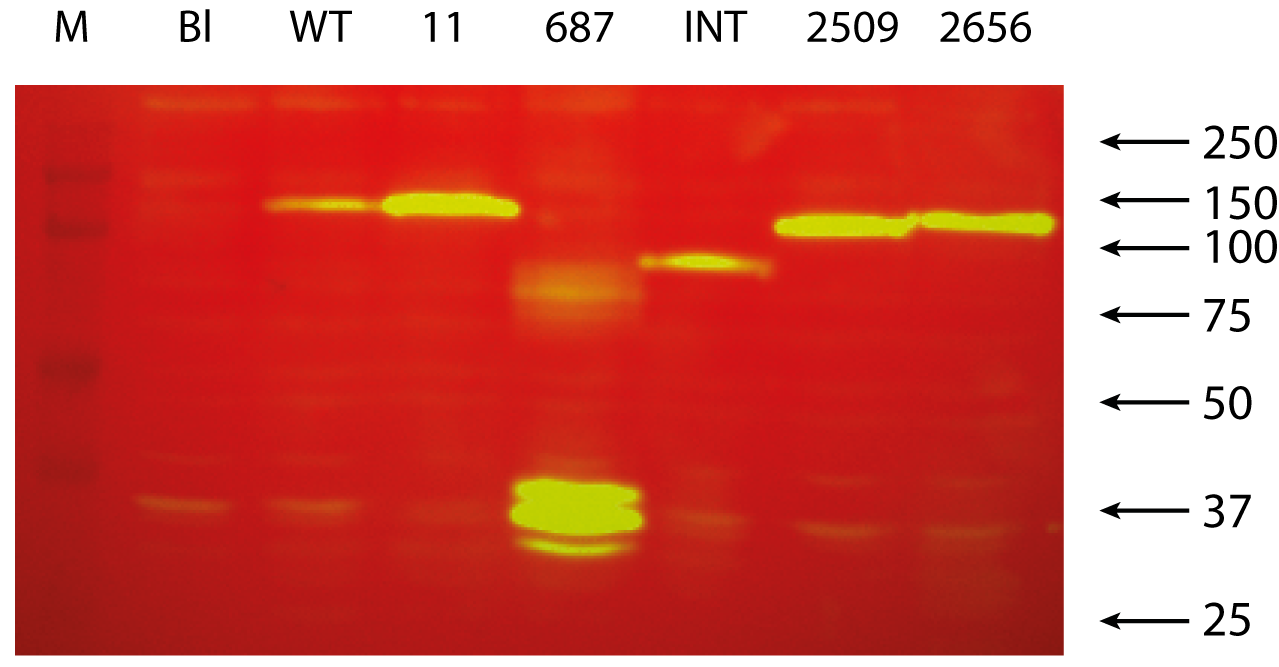

Supplement: Supplementary file 2 — Additional file 2. Protein analysis of wild type and mutant GIIα in HeLa cells. Western blot detection using anti-Flag antibody of cell lysate from transiently transfected HeLa cells using N-terminal Flag tagged construct with GANAB Wild Type (WT) and variants c.11_16delTAGCGG (11), c.687delT (687), c.2002+1G>C (INT), c.2509C>T (2509) and c.2656C>T (2656) at the left of the marker (M). The constructs showed similar amounts of expressed GIIα with predicted molecular weights [file 13023_2020_1585_MOESM2_ESM.tif]

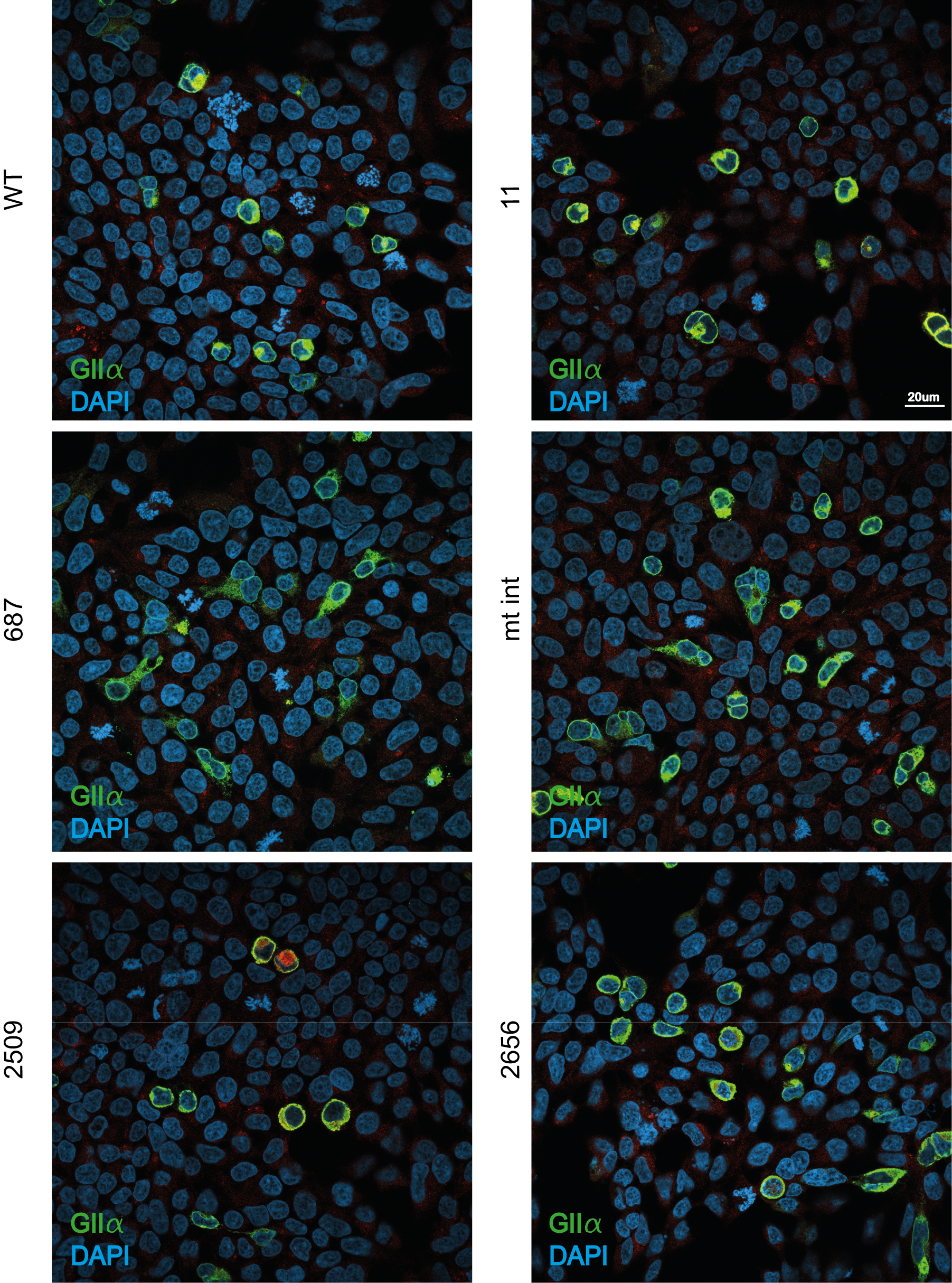

Supplement: Supplementary file 4 — Additional file 4. Localization of wild-type and mutant GIIα in HeLa cells. Immunofluorescence staining of cells transfected with GANAB Wild Type (WT) and variants c.11_16delTAGCGG (11), c.687delT (687), c.2002+1G>C (int), c.2509C>T (2509), and c.2626C>T (2656). GIIα (green) staining shows normal localization of WT, variant 11, variant 2509, and variant 2656. Variant 687 shows subcellular localization. Variant int localizes to the nucleus (blue), or localizes subcellular [file 13023_2020_1585_MOESM4_ESM.tif]
