# Supplementary material for: Novel GANAB variants associated with polycystic liver disease
Source: Orphanet J Rare Dis. 2020 Oct 23;15:302. doi: 10.1186/s13023-020-01585-4 (PMC7585303; doi:10.1186/s13023-020-01585-4)
Supplement: Supplementary file 3 — Additional file 3. DNA and protein expression analysis of GIIα. DNA expression of GIIα Wild Type intron (WT) and mutant intron (MT intron) was analysed using qPCR with B-actin (ACTB) as reference. WT expression was 1.46 times higher than MT intron. On Western Blot expression of WT protein was 4 times higher than MT intron protein. In absolute numbers protein expression of WT intron was 2.67 times higher than MT intron protein [file 13023_2020_1585_MOESM3_ESM.docx]

**Additional File 3** DNA and protein expression analysis of GIIα
DNA expression of GIIα Wild Type intron (WT) and mutant intron (MT intron) was analysed using qPCR with B-actin (ACTB) as reference. WT expression was 1.46 times higher than MT intron. On Western Blot expression of WT protein was 4 times higher than MT intron protein. In absolute numbers protein expression of WT intron was 2.67 times higher than MT intron protein.

| ***WT*** | **Sample** | **Cq** | **Average Ct** | **ΔCt** | **ΔΔCt** | **Difference DNA expression** | **Protein measurement** | **Difference protein measurement** | **Difference DNA/protein expression** |
| --- | --- | --- | --- | --- | --- | --- | --- | --- | --- |
| GANAB F4R4 | 1 | 19.79 | 19.88 | 1.51 | 0.55 | 1.46 | 561568797 | 3.91 | 2.67 |
|  | 2 | 19.89 |  |  |  |  |  |  |  |
|  | 3 | 19.95 |  |  |  |  |  |  |  |
| ACTB | 1 | 21.4 | 21.38 |  |  |  |  |  |  |
|  | 2 | 21.32 |  |  |  |  |  |  |  |
|  | 3 | 21.43 |  |  |  |  |  |  |  |
| ***MT intron*** |  |  |  |  |  |  |  |  |  |
| GANAB F4R4 | 1 | 20.72 | 20.75 | 0.96 |  |  | 143789856 |  |  |
|  | 2 | 20.78 |  |  |  |  |  |  |  |
|  | 3 | 20.74 |  |  |  |  |  |  |  |
| ACTB | 1 | 21.54 | 21.70 |  |  |  |  |  |  |
|  | 2 | 21.88 |  |  |  |  |  |  |  |
|  | 3 | 21.69 |  |  |  |  |  |  |  |
